# Differential effects of ecosystem engineering by the superb lyrebird *Menura novaehollandiae* and herbivory by large mammals on floristic regeneration and structure in wet eucalypt forests

**DOI:** 10.1002/ece3.8956

**Published:** 2022-06-02

**Authors:** Alex C. Maisey, Angie Haslem, Steven W. J. Leonard, Andrew F. Bennett

**Affiliations:** ^1^ 2080 Department of Environment and Genetics La Trobe University Bundoora Vic. Australia; ^2^ 2080 Research Centre for Future Landscapes La Trobe University Bundoora Vic. Australia; ^3^ Department of Primary Industries, Parks, Water and Environment Hobart Tas. Australia

**Keywords:** ecosystem engineer, exclusion experiment, litter and soil modification, lyrebird, herbivory, plant‐animal interactions, seedling germination

## Abstract

Ecosystem engineers that modify the soil and ground‐layer properties exert a strong influence on vegetation communities in ecosystems worldwide. Understanding the interactions between animal engineers and vegetation is challenging when in the presence of large herbivores, as many vegetation communities are simultaneously affected by both engineering and herbivory. The superb lyrebird *Menura novaehollandiae*, an ecosystem engineer in wet forests of south‐eastern Australia, extensively modifies litter and soil on the forest floor. The aim of this study was to disentangle the impacts of engineering by lyrebirds and herbivory by large mammals on the composition and structure of ground‐layer vegetation. We carried out a 2‐year, manipulative exclusion experiment in the Central Highlands of Victoria, Australia. We compared three treatments: fenced plots with simulated lyrebird foraging; fenced plots excluding herbivores and lyrebirds; and open controls. This design allowed assessment of the relative impacts of engineering and herbivory on germination rates, seedling density, vegetation cover and structure, and community composition. Engineering by lyrebirds enhanced the germination of seeds in the litter layer. After 2 years, more than double the number of germinants were present in “engineered” than “non‐engineered” plots. Engineering did not affect the density of seedlings, but herbivory had strong detrimental effects. Herbivory also reduced the floristic richness and structural complexity (<0.5 m) of forest vegetation, including the cover of herbs. Neither process altered the floristic composition of the vegetation within the 2‐year study period. Ecosystem engineering by lyrebirds and herbivory by large mammals both influence the structure of forest‐floor vegetation. The twofold increase in seeds stimulated to germinate by engineering may contribute to the evolutionary adaptation of plants by allowing greater phenotypic expression and selection than would otherwise occur. Over long timescales, engineering and herbivory likely combine to maintain a more‐open forest floor conducive to ongoing ecosystem engineering by lyrebirds.

## Graphical abstract

Ecosystem engineers that modify the soil and ground‐layer properties exert a strong influence on vegetation communities in ecosystems. Using an experimental approach, we showed that foraging by the superb lyrebird (*Menura novaehollandiae*), an avian engineer in Australian eucalyptus forests, resulted in a nearly twofold increase in seeds stimulated to germinate, while large mammalian herbivores reduced plant species richness. Ecosystem engineering by lyrebirds may contribute to the evolutionary adaptation of plants by allowing greater phenotypic expression and selection than would otherwise occur.

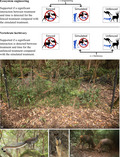

## 1 INTRODUCTION

Ecosystem engineers play important roles in ecosystems worldwide (Wright & Jones, 2006). Vertebrates, particularly mammals, that excavate soils when foraging or constructing burrows have received much attention for their impacts on soils, especially in arid environments (Romero et al., 2014; Whitford & Kay, 1999). Such soil manipulation alters plant communities through both direct and indirect pathways (Fleming et al., 2014; Valkó et al., 2021). Digging animals can directly destroy individual plants (Song et al., 2012) while enhancing the spread of seeds through caching (Murphy et al., 2005) or epizoochory (Rodrigues et al., 2020; Wilby et al., 2001). Soil displacement alters the chemical and structural properties of soils, increases water infiltration (Garkaklis et al., 1998), run‐off and erosion (Eldridge & Myers, 2001), and moderates the availability of soil nutrients such as labile carbon, nitrogen, and sulfur (Eldridge & Mensinga, 2007). Conditions within foraging pits can provide a microclimate conducive to plant germination (Eldridge & Koen, 2021; Louw et al., 2021; Martin, 2003) while burying and mixing of litter and soil allows greater assimilation by macro‐ and micro‐invertebrates, thus fuelling high rates of nutrient cycling (Mallen‐Cooper et al., 2019; Valentine et al., 2013) and improving plant growth (Fleming et al., 2014; Valentine et al., 2018).

The role of birds as ecosystem engineers has received limited attention (Coggan et al., 2018; Sekercioglu, 2006), although growing evidence suggests that many species moderate habitats in ways that affect vegetation (Bancroft et al., 2005; El‐Hacen et al., 2019; Sekercioglu et al., 2016). For example, the excavation of nesting burrows and deposition of guano by the sooty shearwater *Puffinus griseus* on off‐shore islands of New Zealand combine to profoundly alter vegetation communities around their large breeding colonies (McKechnie, 2006). Disturbance to leaf litter by ground‐foraging birds such as the malleefowl *Leipoa ocellata* (Smith et al., 2017), Australian brush‐turkey *Alectura lathami* (Song et al., 2012), and greater bowerbird *Chlamydera nuchalis* (Mikami et al., 2010) can moderate vegetation structure, increase fine‐scale habitat heterogeneity, and affect ecological processes. Such ground‐foraging species can physically destroy small plants, but their activities may also promote conditions for germination.

Understanding the interactions between animal engineers and vegetation can be challenging when in the presence of large herbivores, because many vegetation communities may be simultaneously affected by both engineering (non‐trophic, physical modification) and herbivory (trophic effects from browsing, grazing). The effects of herbivores on plant communities, especially when overabundant, may mask those of ecosystem engineers. Alternatively, the activities of engineers may ameliorate the potentially negative effects of herbivores. The interactions between engineers and large herbivores have rarely been addressed, yet such understanding is likely to be instructive for conservation management (Wilby et al., 2001).

The superb lyrebird *Menura novaehollandiae*, a large (0.7–1.2 kg), ground‐foraging bird, is an ecosystem engineer in moist eucalypt forests in south‐eastern Australia, owing to its ability to move vast amounts of litter and soil (Ashton & Bassett, 1997; Maisey et al., 2021) when foraging for invertebrate prey. As lyrebirds work through a forest stand, their scratching and foraging (hereafter ‘engineering’) create micro‐habitats and niche opportunities for ground‐layer plants, in the form of discrete litter piles interposed with bare soil, litter‐free pits, and micro‐terraces (Ashton & Bassett, 1997), all arranged within a matrix of leaf litter covering the soil. Furthermore, a suite of non‐plant organisms potentially takes advantage of such spatial heterogeneity (Hansen, 2000), including bacteria and fungi (Eldridge et al., 2016), micro‐ and macro‐invertebrates, and detritivore predators such as arachnids (Bultman & Uetz, 1982; Langellotto & Denno, 2006), thereby affecting soils and hence vegetation through multiple pathways. Several large herbivores are also present in these moist eucalypt forests, including the swamp wallaby *Wallabia bicolor*, common wombat *Vombatus ursinus* and the introduced sambar deer *Rusa unicolor*. Browsing and grazing by these herbivores are likely to interact with engineering effects by lyrebirds, to modify the vegetation.

**FIGURE 1:**
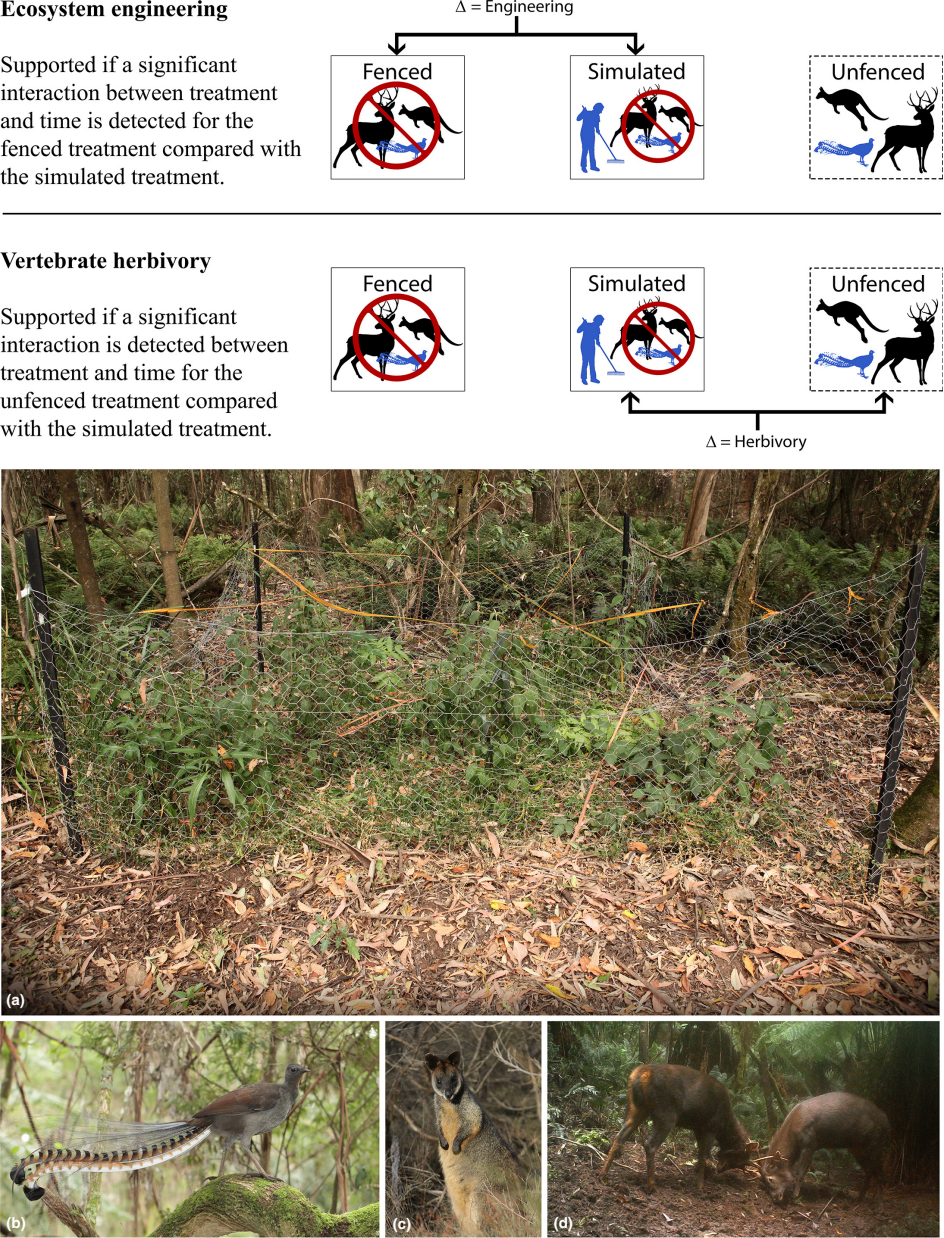
Schematic diagram representing the three experimental treatments at each site. Fenced plots exclude habitat modification by lyrebirds and herbivory by vertebrates. Simulated plots are fenced and have a monthly simulation of lyrebird engineering, undertaken by hand‐raking. Unfenced plots remain accessible to lyrebirds and vertebrate herbivores. The “∆” defines which effect is tested by comparisons between treatments. Note that both hypotheses are tested simultaneously with each test performed. Also pictured are examples of (a) a fenced plot after 24 months, (b) a male superb lyrebird, (c) a native swamp wallaby, and (d) two introduced sambar deer stags

We used a manipulative exclusion experiment to evaluate the impact of engineering by the superb lyrebird, both in isolation and combined with herbivory, in three forest types in wet forests of south‐eastern Australia. To disentangle the effects of engineering from those of herbivory on plant communities, we established three treatments: (a) “fenced,” where lyrebirds and large herbivores were excluded; (b) “simulated,” where lyrebirds and herbivores were excluded but lyrebird engineering activity was simulated; and (c) “unfenced,” where both engineering by lyrebirds and herbivory from large herbivores could occur. By comparing the simulated treatment with the fenced treatment, the impact of engineering was isolated from that of herbivory; and by comparing the simulated treatment with the unfenced treatment, the effect of herbivory could be evaluated (Figure 1).

We hypothesized that lyrebird engineering and mammalian herbivory would both alter plant community composition while having opposing effects on species richness. We predicted that floristic richness would increase with engineering, consistent with the expectation that the engineered structures (e.g., foraging pits) would create niche opportunities for plants (Wilby et al., 2001); whereas richness would decrease with herbivory (Fuller & Gill, 2001; Parsons et al., 2007) owing to the removal of plants at the plot scale. The structural complexity of low‐growing vegetation (measured by the cover of herbs and ground ferns, and vegetation contacts in height intervals) was expected to be maintained at a constant (low) level as a result of lyrebird disturbance. Finally, we predicted that lyrebird engineering would promote the germination of seeds by clearing leaf litter, disturbing the topsoil, and exposing buried seeds to light; while at the same time decreasing the survivorship of many growing seedlings due to uprooting or burial. In contrast, herbivory was expected to have little influence on germinants (due to their small size and fast growth), while decreasing the number of larger seedlings.

## 2 METHODS

### 2.1 Study area

**FIGURE 2:**
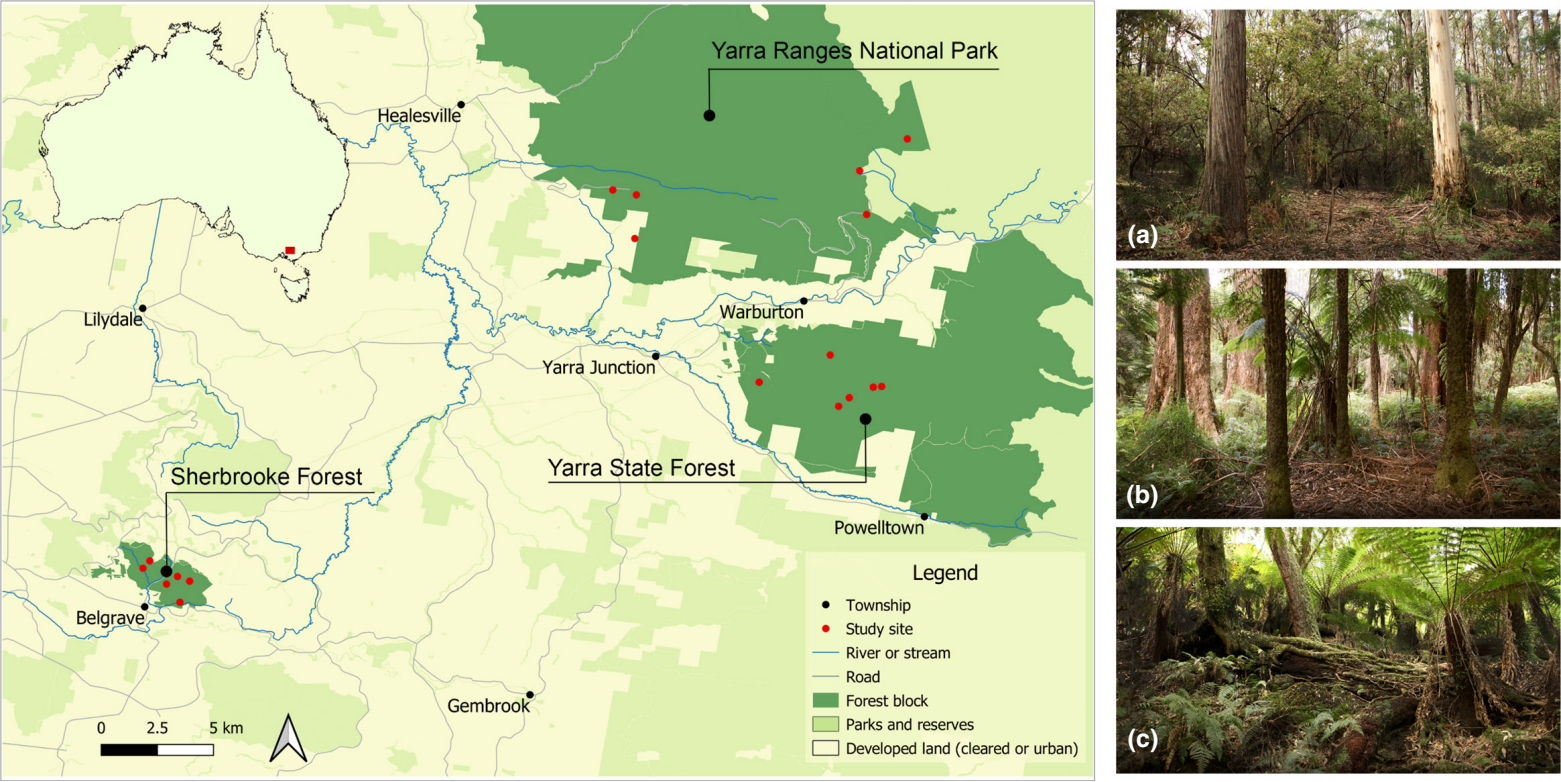
The study region shows three forest blocks and the location of study sites within each. The panel at the right provides exemplar images of (a) damp forest, (b) wet forest, and (c) cool temperate rainforest

This study was conducted in the southern fall of the Central Highlands of Victoria, Australia (Figure 2). The topography is characterized by moderate to steep slopes and high plateaus; valleys are comprised of alluvial flats. Three forest blocks were selected: (1) Sherbrooke Forest; (2) Yarra Ranges National Park; and (3) Britannia Creek (Figure 2). Forest blocks were geographically isolated by >10 km of mostly semi‐rural land. Experimental manipulations were undertaken within each forest block (Maisey et al., 2021).

In this region, lyrebirds commonly occur in three distinct forest types: cool temperate rainforest, wet forest, and damp forest (Loyn, 1985; van der Ree & Loyn, 2002). The cool temperate rainforest is dominated by southern sassafras *Atherosperma moschatum* and myrtle beech *Nothofagus cunninghamii* (though the latter is absent in Sherbrooke Forest), with a fern‐rich understorey. Typically, the ground layer is more open than the other forest types due to shading from the dominant tree species and abundant soft tree ferns *Dicksonia antarctica*. Shade‐tolerant herbs (e.g., shade nettle *Australina pusilla* and forest pennywort *Hydrocotyle geraniifolia*) are common groundcovers, as are colonial ground ferns (e.g., hard water fern *Blechnum watsii* and mother‐shield fern *Polystichum proliferum*) which usually occur in loose colonies, owing to the low‐light environment.

The wet forest is widespread, much of which is the regrowth mountain ash *Eucalyptus regnans* that regenerated following severe bushfires in 1939. Wet forest typically has a tall eucalypt canopy, with a multi‐structured mid‐story of blackwood *Acacia melanoxylon* and silver wattle *Acacia dealbata* over a diverse mix of small trees. The ground layer is a patchwork of colonial ground ferns in a loose matrix of open leaf litter. Soft tree ferns and rough tree ferns *Cyathea australis* are also widespread.

Damp forest is dominated by messmate *Eucalyptus obliqua* and mountain gray gum *Eucalyptus cypellocarpa*. The mid‐story, where present, is similar in composition to wet forest; the ground layer has Austral bracken *Pteridium esculentum*, sedges such as *Lepidosperma elatius* and *Gahnia sieberiana*, and a diverse herb layer, occasionally mixed with grasses (e.g., weeping‐grass *Microlaena stipoides* and forest wire grass *Tetrarrhena juncea*). The dominant ground fern is gristle fern *Blechnum cartilagineum*, which may form dense colonies on moist slopes.

The superb lyrebird and the three large herbivores are ubiquitous in all three forest blocks (van der Ree & Loyn, 2002). They forage in each forest type (Maisey et al., 2019), but lyrebirds typically avoid forest that has recently experienced wildfire (Nugent et al., 2014) and prefer to forage in areas with open ground cover (Ashton & Bassett, 1997), and thus are likely to avoid young regrowth forest recovering from fire or logging. Each forest block contains mature stands of the three forest types and has not undergone logging or experienced severe wildfire for >50 years.

### 2.2 Experimental design

In each forest block, potential sites in each of the three forest types were compiled using random coordinates (Maisey et al., 2021). In total, 18 sites were selected (i.e., three forest blocks X three forest types X two replicates in each forest type).

At each of the 18 sites, three experimental plots (3 × 3 m) were established in October 2015. Plots were positioned along the contour of the slope, with each plot placed at a random distance between 5 and 40 m from one another. Vegetation was surveyed in the field before plot establishment to ensure the cover of low vegetation (<50 cm) would not inhibit foraging by lyrebirds. Counts of contacts with a vertical pole were used to ensure no more than five of nine point‐measures (arranged in a cross‐centered on each plot) intersected with vegetation, as lyrebirds avoid foraging in vegetation beyond this density (Maisey et al., 2019). If the vegetation at the first randomly selected distance was too structurally complex, the contour was followed until the vegetation was sufficiently open and each plot conformed. One of the three plots was randomly assigned as a reference plot, to remain unfenced and accessible to lyrebirds and herbivores. The two remaining plots were fenced to exclude lyrebirds and herbivores; one remained undisturbed, and the other was subject to simulated lyrebird engineering (see below) on monthly visits. Fences were constructed of wire netting with 5 cm mesh size, a height of 120 cm, and pinned at ground level. There was no evidence of fence effects on litter depth extending beyond 20 cm into fenced plots (Maisey et al., 2021). The flagging tape was strung across the top of the fence to deter lyrebirds from flying into the plot (Figure 1a).

During November 2015 and August to October 2016, a single motion‐sensing camera (Reconyx hyperfire, model HC600) was set at the reference (unfenced) plot at each site to confirm the presence of lyrebirds and herbivores. Cameras were programmed to capture two images per trigger event, with a 60 s rest period. Large herbivores were widespread across the study region, and included sambar deer (16 sites), swamp wallaby (16 sites), and common wombat (12 sites). Lyrebirds were confirmed present at every site (18 sites).

Fences were monitored for damage on a monthly basis. At a small proportion of monthly checks (6 plots, representing <3% of all observations), fences were thought to have been breached by lyrebirds, particularly during the first 6 months when fences were new to the environment. If diggings consistent with that of a lyrebird were evident in a fenced plot it was recorded, though this was so seldom observed that effects were assumed to be negligible. No evidence of browsing was detected within plots; hence, herbivores were not thought to have breached the fences at any stage.

### 2.3 Simulation of lyrebird litter and soil engineering

On each monthly visit to a site, the area disturbed by lyrebirds in the unfenced plot was visually assessed and recorded as a percent cover estimate. In the fenced simulated plot at that site, foraging activity (engineering) was then simulated using a three‐pronged hand rake (the approximate width of a lyrebird foot, ~10 cm), to replicate as closely as possible the cover and configuration of engineered litter and soil recorded in the unfenced plot (lyrebird disturbance of litter and soil in unfenced plots ranged from 0% to 100% of the plot area, with a monthly mean of ~11%). A comparison of litter and soil properties between treatments (Maisey et al., 2021) showed that simulated and unfenced plots were similar; and both differed significantly from fenced plots in relation to soil compaction and litter depth. Nevertheless, simulated engineering may not mimic natural lyrebird foraging in all aspects.

### 2.4 Data collection

All vascular plant species were surveyed in each 3 m × 3 m plot during baseline sampling in October 2015, then at 12‐month intervals for the 2‐year experiment (i.e., 3 sample periods in total). The total number of plant species (floristic richness) was summed at each visit. No attempt was made to identify germinating species, due to difficulty in distinguishing between similar species.

All germinant dicotyledonous plants (i.e., at first emergence, no true leaves), hereafter “germinants,” were counted within a 1 × 1 m subplot, centered on each plot. The subplot was used to avoid the potential influence of edge effects introduced by the fences interacting with litter (see below). Germinant counts were recorded upon plot establishment (baseline, Oct 2015), and then at three‐monthly intervals for 2 years (i.e., 9 sample periods). From the same 1 × 1 m subplot, the number of small dicotyledon plants that had only true leaves and a stem diameter <10 mm, was recorded, hereafter referred to as “seedlings.”

Concurrent with three‐monthly surveys, vegetation structure was measured using a modified structure‐pole technique (Chaffey & Grant, 2000). A 2 m pole was placed vertically and the presence or absence of vegetation touches (dead or alive), were recorded in 50 cm increments from ground level to 2 m. This procedure was repeated at five points (at the center and four corners of the 1 × 1 m subplot) in each plot. Cover estimates (% cover) of ground ferns and herbs were visually assessed (by a single observer, AM) for the entire 3 × 3 m plot. Grasses were included in the measure of herb cover because these occupy the same strata as herbs and were rarely recorded in plots.

### 2.5 Statistical analysis

All statistical analyses were implemented in the R programming language (R Core Team, 2012) using the R Studio interface (RStudio, 2012).

Change in floristic composition through time was investigated using the “vegan” package (Oksanen et al., 2013). PERMANOVA tests were conducted using the “adonis” function, with the Bray–Curtis dissimilarity measure, based on the presence or absence of vascular plant species, excluding epiphytes, tree ferns, and canopy tree species (*Eucalyptus*, *Acacia*), as these are large, long‐lived species, assumed to not be affected by the treatments over this 2‐year study. Of key interest was the interaction between treatment and time (3 sample periods). Forest type was also included as an explanatory variable. “Adonis” was set to carry out permutations within “forest block” using the “strata” argument to account for spatial patterns associated with sampling within different forest blocks. Within‐group homogeneity of variance (dispersion) was tested for each time period and treatment using the “betadisper” function. There was no difference in group variances (dispersion) between treatments or time periods (betadisper *p *> .05), indicating assumptions for PERMANOVA were met (Oksanen et al., 2013). To visualize the results, we generated an ordination using non‐metric multi‐dimensional scaling (NMDS) for the dataset at 24‐months (i.e., end of the experiment): the ordination was visualized using the “metaMDS” function.

To model the effect of the experimental treatments on floristic richness, we used a linear mixed model (LMM) with a Gaussian distribution to assess change between treatments through time (included as a continuous interaction term in the model to account for lack of independence). The same long‐lived plant species were excluded, as above. Floristic richness was log‐transformed to improve normality. The interaction between treatment and time, and forest type, were specified as fixed effects and a random term was included that nested “plot” within “site” and “forest block,” to account for repeat measures through time and the spatial clustering of plots.

Counts of germinants and seedlings were each modeled using a generalized LMM (GLMM) assuming a Poisson distribution. In each model, the interaction between treatment and time was included, with the simulated treatment as the reference category to allow for explicit comparison between engineering effects (i.e., simulated cf. fenced plots) and herbivory effects (i.e., simulated cf. unfenced plots). Forest type and season were also included in each model. A nested random term was specified, for the LMM above.

Vegetation structure was analyzed with logistic regression (GLMM), assuming a binomial distribution, with the response variable being the proportion of vegetation touches from five samples (measured as the presence or absence) for each height interval (0–0.5, 0.5–1.0, 1.0–1.5, and 1.5–2.0 m) for each plot. Cover estimates (% cover) of ground ferns and of herbs were logit‐transformed, as this transformation is suitable for percentage data (Warton & Hui, 2011), and modeled with an LMM assuming a Gaussian distribution. Forest type and the interaction between treatment and time were included as fixed effects and a nested random term was included as described above.

All linear models were constructed using the package “lme4” (Bates et al., 2014); model predictions were generated with the package “Effects” (Fox, 2003) and visualized using the package “ggplot2” (Wickham, 2011).

## 3 RESULTS

### 3.1 Floristic composition

Floristic composition differed between forest types (adonis test, *p *< .01) but there was no evidence of a significant interaction between treatment and time (*p *= .99; Appendix A). Thus, during the final sampling period (24‐months), the NMDS ordination showed no evidence of a clear response to experimental treatments, but there was clustering of sites by forest type (Appendix B).

### 3.2 Floristic richness

**FIGURE 3:**
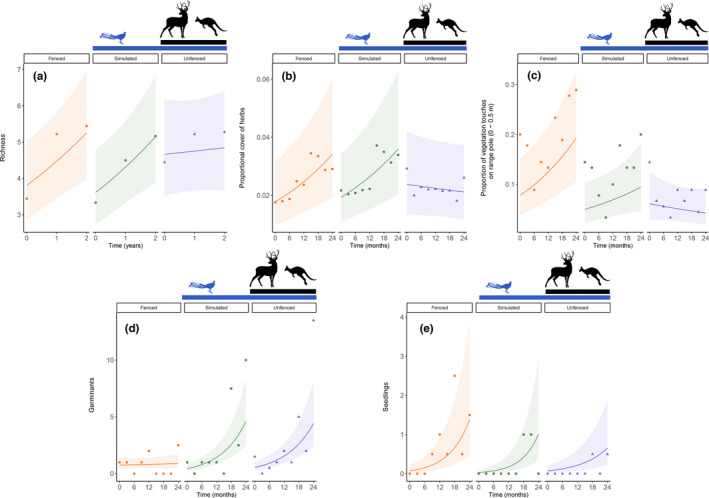
Model prediction plots (±95% C.I.s) for (a) a linear mixed model of floristic species richness through time; (b) generalized linear mixed model of changes in low vegetation structure (touches on ranging pole, 0–0.5 m) through time; (c) linear mixed model for changes in herb cover through time; and generalized linear mixed models of changes in (d) germinants and (e) seedlings through time for each of the three treatments. Data points that show mean (a–c) or median (d–e) values for each 3‐monthly count are overlayed (medians are used to best represent data with a Poisson distribution). For each model, the three treatments are shown as separate panels for clarity

Measures of plant species richness (excluding epiphytes, tree species, and tree ferns) increased through time in all treatments. Baseline (Oct 2015) richness measures were a mean of 3.3 species in simulated, 3.4 in fenced, and 4.4 in unfenced plots; and increased through the study to mean values of 5.2, 5.4, and 5.3 species, respectively, at the final sampling period (24 months). There was a significant interaction between unfenced and simulated treatments through time (Appendix C). That is, there was a much lower increase in richness for the unfenced compared with the simulated plots (Figure 3a), although both experienced similar soil disturbance. This difference can be attributed to the effects of herbivory on vegetation in unfenced plots, whereas herbivores were excluded from simulated plots. Species richness in fenced plots showed a similar trend through time to simulated plots (Figure 3a), with no interaction between treatment and time. Thus, engineering by lyrebirds did not affect floristic richness during the 2‐year period.

### 3.3 Germinants

The number of germinants (emergent dicotyledons with no true leaves) increased through time in all treatments, with median baseline (Oct 2015) counts of 1 germinant in fenced, 1 in simulated, and 1.5 in unfenced treatments (mean values, 2.8, 4.3, and 5.1 germinants, respectively), increasing to 2.5 in fenced, 10 in simulated, and 13.5 in unfenced treatments (means 7.3, 15.0, and 22.4, respectively) by the final count at the end of the 2‐year period. There was a significant interaction between treatments and time (Appendix C). The number of germinants showed little increase through time in fenced plots but increased strongly in simulated plots over the duration of the experiment (Figure 3d). This difference represents a positive effect of engineering on germinants. Conversely, there was no significant interaction between unfenced and simulated treatments through time: in both treatments the numbers of germinants increased strongly, suggesting potential herbivory (in unfenced plots) had little impact on germinant counts. There also was a seasonal effect: more germinants were recorded in spring and fewer in summer, than in autumn (reference category) (Appendix C).

### 3.4 Seedlings

Models showed that counts of seedlings (dicotyledons with true leaves and stem <10 mm) increased in all treatments during the study, with median baseline counts of 0 seedlings for all treatments (mean counts, 0.2 seedlings in all treatments). By the end of the study, median seedling counts had increased to 1.5 seedlings in fenced treatments, 0.5 in simulated treatments, while remaining at 0 for unfenced treatments (mean counts of 5.5, 4.2, and 2.2 seedlings, respectively). A significant interaction was identified between unfenced and simulated plots through time. There was a greater increase in seedlings in simulated plots compared with unfenced plots (Figure 3e), representing an effect of herbivory on seedlings, with herbivores able to access unfenced plots but not those with simulated engineering. There was no interaction between simulated and fenced treatments through time; both showed a similar increase in seedling counts compared with the unfenced treatment (Figure 3e). There also was a significant seasonal effect, with greater numbers of seedlings counted in autumn than in the other three seasons (Appendix C).

### 3.5 Vegetation structure

Vegetation structure in the lowest height interval (0–0.5 m) showed an interaction between treatment and time for unfenced compared with simulated plots (Figure 3c). There was a greater increase in structure (contacts with vertical pole) through time in simulated plots than in unfenced plots, consistent with herbivory by vertebrates in the unfenced plots reducing low vegetation structure. No interactions were present for any of the higher height strata modeled (Appendix D).

### 3.6 Cover of herbs and ground ferns

Model outputs revealed that herb cover increased through time in fenced and simulated treatments but decreased in unfenced treatments (Appendix E). A small but significant interaction was detected between simulated and unfenced plots, indicative of vertebrate herbivory reducing the cover of herbs in unfenced plots. The trajectory of increasing herb cover through time in simulated and fenced treatments did not differ (i.e., no engineering effect; Figure 3b). Ground fern cover did not change during the study period, with no significant treatment by time interactions (Appendix E).

## 4 DISCUSSION

Recognition of the complex interactions between herbivores and ecosystem engineers in ecosystems has increased in recent years (Parker et al., 2007; Wilby et al., 2001), but field studies that explicitly distinguish their relative impacts remain uncommon. While challenging, disentangling trophic impacts of herbivory from those of engineering can provide nuanced insights into the dynamics of plant communities and the potential for flow‐on effects on other components of the biota (Grinath et al., 2018; Prugh & Brashares, 2012). In this manipulative experiment, our capacity to distinguish the trophic effects of vertebrate herbivores from the engineering effects of lyrebirds gave insights into processes influencing plant communities in wet eucalypt forests. Lyrebird engineering exerted a strong influence on the abundance of germinants in plots. Herbivory had a strong influence on floristic richness, the number of seedlings, vegetation structure in low strata (<50 cm) and herb cover; attributes that were not directly related to the engineering of litter and soil by lyrebirds. Other attributes of the plant community, including the floristic composition of the vegetation and cover of ground ferns, showed no evidence of the direct influence of either engineering or herbivory.

### 4.1 Effects of herbivory on the plant community

Vertebrate herbivores often have neutral or negative impacts on vegetative growth in natural systems (Fuller, 2001; Travers et al., 2018), but may have positive effects on floristic richness via the suppression of competitively dominant plant species (Collins et al., 1998). During the 2 years of this study, however, vertebrate herbivory did not increase richness but rather showed the opposite trend: when herbivores (e.g., swamp wallaby and sambar deer) were excluded by fencing, the richness of plant species increased. Understorey shrubs and small plants that are palatable to herbivores, such as *Coprosma quadrifida*, *A*. *pusilla*, *M*. *stipoides*, and *T*. *juncea*, frequently became established in fenced treatments, while showing little change in the unfenced treatment. Vertebrate herbivory also appeared to suppress the amount of low strata vegetation (<50 cm height) in the unfenced treatment. A similar pattern was evident for herb cover, with an increase in cover in simulated and fenced treatments, but not the unfenced treatment. These effects likely reflect differential levels of browsing on plant species by potentially overabundant herbivores (Royo et al., 2017), resulting in patchy local suppression of palatable species (Foster et al., 2016).

Seedling counts also were influenced by herbivory. At the end of the study there were fewer seedlings in the unfenced treatment, but no difference between fenced and simulated treatments. Interpreting this effect, however, is not straightforward. In the fenced treatments, engineering effects of lyrebirds were present when fences were constructed: that is, lyrebirds had “primed” the ground layer for germination (i.e., a broken litter layer and exposed seed bank). In the absence of herbivores (excluded by fencing), a flush of growth occurred within these treatments. These survived to reach the seedling stage during the study, resulting in a legacy effect of lyrebirds reflected in our results. In simulated treatments, while some seedlings were likely removed or smothered by simulated engineering actions, this attrition was compensated by new seedlings from seeds stimulated to grow by the same process. Germinants, however, were not affected by herbivory, likely due to their small size conferring limited nutritional benefits to large‐bodied herbivores and hence being largely overlooked.

### 4.2 Effects of engineering on the plant community

Given the massive scale of soil and litter displacement by lyrebirds, a mean of 155 tonnes/ha per year (Maisey et al., 2021), it is surprising that engineering effects on vegetation were not more prominent. With the displacement of litter and soil across an average of ~11% of unfenced plots per month (Maisey et al., 2021), such that areas suitable for foraging may be entirely turned over in less than a year, it could be expected that marked changes would occur to the structure and composition of ground‐layer vegetation. We suggest that the apparently limited or neutral effects of such extensive engineering reflect compensatory mechanisms that allow vegetation to respond rapidly to disturbance. For example, engineering by lyrebirds neither increased nor decreased plant species richness (i.e., simulated treatments did not differ from fenced treatments). Many common herb species in wet forests are small and likely to be uprooted or displaced by lyrebirds (e.g., shade nettle *A*. *pusilla*, ivy‐leaf violet *Viola hederacea*) but also are fast‐growing, with germination potentially stimulated by altered soil conditions. We suggest that, over the long term, local disturbance and removal of species through foraging activities, together with suppression or elimination of some species by herbivores, may be compensated by the creation and maintenance by lyrebirds of niche opportunities that are exploited by other plant species, thus maintaining local richness on the forest floor.

Counts of germinants provided evidence of a strong effect of ecosystem engineering by lyrebirds. In both the simulated and unfenced treatments (i.e., both subjected to engineering effects), the number of germinants increased through time, while in fenced plots there was little change. The increase in germinant counts in all treatments (particularly simulated and unfenced plots) through the 2‐year study is most likely attributable to increased rainfall. Rainfall across the study region was as much as ~30% below average during and in the year preceding the first season of the study, while in the second and third seasons rainfall increased to 95%–110% of the long‐term average (Bureau of Meteorology, 2019). Concomitant with wetter conditions, litter decomposition rates increased leading to a shallower litter layer (Maisey et al., 2021), and reduced litter‐smothering of seeds and seedlings. As a result, fenced plots (without engineering) underwent little change in litter depth and germinant counts, rather than germinant counts decreasing in response to accumulating litterfall as initially predicted.

When lyrebirds forage, they scrape the litter layer and mix and bury much of the surface litter with mineral soil. This disturbance decreases litter depth (Maisey et al., 2021), creates gaps in the litter layer, and potentially promotes light‐driven germination, especially important for small seeds of many temperate forest species (Theimer & Gehring, 1999). Further, by creating a finely heterogeneous litter profile, the variation in depth and extent of mineral soil mixing may support the germination of a wider range of species that require specific litter conditions for germination and growth (Green, 1999).

There was no evidence of either engineering or herbivory affecting ground ferns. These species (e.g., *P*. *proliferum* and *Blechnum wattsii*) are much larger than herbs and longer‐lived. At high density, ground fern cover does inhibit foraging by lyrebirds (Maisey et al., 2019). Over a longer timescale, we predict that foraging by lyrebirds will slow or prevent widespread colonization by ground ferns, by the physical destruction of young, asexually reproduced plantlets, and the uprooting of young rhizomatous species. Anecdotal evidence of dead, uprooted juveniles of mother‐shield fern *Polystichum bulbiferum* on the fringe of dense fern colonies, suggests lyrebird activity maintains the patchwork structure of open litter areas between ground fern colonies. Interactions between lyrebirds and ground ferns are thus likely to determine forest understorey patterns over prolonged timescales; however, our 2‐year study was not sufficiently prolonged to examine these interactions.

### 4.3 Implications for ecological processes in wet forests

Interactions between engineering by lyrebirds and the germination and survivorship of seedlings have implications for the evolutionary potential of plants in wet eucalypt forests. Two primary pathways are possible. First, in the presence of lyrebirds, the number of seeds afforded the opportunity to germinate is higher, stimulated by exposure to light and mechanical abrasion/disturbance (Clarke et al., 2000; Floyd, 1976), thereby allowing the establishment of more individuals of more species. In turn, greater phenotypic diversity will be expressed in a highly competitive environment, over time facilitating higher fitness by retaining beneficial genes that may otherwise be rarely expressed in plant populations. Second, through burying or partial uprooting of seedlings as they grow, such extensive engineering of litter and soil may select for particular phenotypes in plant populations. For example, seedlings with strong, fast‐growing roots and higher tolerance to water stress will be more resistant to antagonistic disturbances. Both pathways may increase the resilience of plant communities to disturbance and environmental extremes. Should lyrebirds be lost from wet forest ecosystems, plants may be less‐adapted for germination in the litter‐rich environment of the forest floor.

Ecosystem engineering by lyrebirds also potentially interacts with wildfire, a profoundly important process that shapes the dynamics of wet eucalypt forests (Bowman et al., 2020; Fairman et al., 2016; Lindenmayer et al., 2019). By burying and mixing litter with soil, lyrebirds reduce fuel loads and create conditions for wildfire to burn at a lower intensity (Nugent et al., 2014), thereby indirectly influencing post‐fire vegetation recovery. Second, the extent of lyrebird foraging after a severe wildfire is likely to shape the pattern of vegetation recovery. Following severe wildfire, lyrebird populations occur at lower density or may be absent in early successional stages (Nugent et al., 2014).

## 5 CONCLUSIONS

Ecosystem engineering by lyrebirds through the turnover of the soil and litter layer, and herbivory by large mammalian herbivores, both have a distinct influence on the structure of plant communities in the extensive wet eucalypt forests of southern Australia. Herbivory suppressed seedling survivorship, herb cover, and vegetation structure of low strata. Engineering strongly enhanced seed germination. Importantly, despite the massive extent of lyrebird disturbance to the forest floor, such engineering did not alter the floristic composition or richness of the vegetation over the 2‐year period of study. The individual and interactive effects of lyrebirds and herbivores are likely to structure wet forest plant communities over long timescales, although longer‐term studies are needed to resolve these processes. Enhanced seedling germination as a consequence of lyrebird engineering may serve as an evolutionary driver of the fitness of understorey plants in these forests.

## AUTHOR CONTRIBUTIONS

**Alex C. Maisey:** Conceptualization (equal); Formal analysis (lead); Methodology (lead); Writing – original draft (lead). **Angie Haslem:** Conceptualization (equal); Formal analysis (supporting); Supervision (supporting); Writing – review & editing (supporting). **Steven W. J. Leonard:** Conceptualization (equal); Formal analysis (supporting); Supervision (supporting); Writing – review & editing (supporting). **Andrew F. Bennett:** Conceptualization (equal); Methodology (supporting); Supervision (lead); Writing – original draft (supporting); Writing – review & editing (supporting).

## CONFLICT OF INTEREST

AM, AFB, and SL conceived the ideas and designed the experiment; AM and AH analyzed the data; and AM and AFB led the writing of the manuscript. All authors contributed critically to the drafts and gave final approval for publication. The authors declare no conflicts of interest.

## Data Availability

Data are available from Dryad Digital Repository: https://doi.org/10.5061/dryad.cjsxksn7v.

## APPENDIX A

Summary of the PERMANOVA test (using Adonis) comparing floristic composition between experimental treatments over time. Significant effects are shown in bold. “*” denotes the interaction term

**Table:**

Factor	df	SumsOfSqs	MeanSqs	*F*	*R* ^2^	*p*
Treatment	2	0.64	0.32	1.10	0.01	.26
Time	1	0.27	0.27	0.91	<0.01	.43
**Forest type**	**2**	**8.67**	**4.33**	**14.84**	**0.17**	**<.01**
Treatment*Time	2	0.16	0.08	0.28	<0.01	.99
Residuals	143	41.75	0.29			

## APPENDIX B

Non‐metric multidimensional scaling ordination comparing floristic composition at experimental plots in the final (24‐month) sample period. The ordination is labelled by (a) treatment and (b) forest type (note: several points are overlapping; ordination stress value = 0.13)

## APPENDIX C

Model outputs for the relationship between experimental treatments over time and (a) floristic richness (LMM assuming a Gaussian error distribution), (b) germinant counts, and (c) seedling counts (GLMMs assuming a Poisson error distribution). For all models, the reference category for treatment was “simulated,” to allow for tests of engineering effects (i.e., simulated c.f. fenced) and herbivory effects (i.e., simulated c.f. unfenced). The reference category for forest type was “Damp forest.” “*” denotes interactions between fixed effects. Significant effects are shown in bold

**Table:**

Response	Fixed effect	Estimate	SE	*t* value	*R* ^2^_(marginal)_	*R* ^2^_(conditional)_
(a) Floristic richness (Log_e_‐transformed)	(Intercept)	1.73	0.20	8.50	0.31	0.82
	**Time**	**0.18**	**0.05**	**3.36**		
	Treatment—Fenced	0.05	0.16	0.33		
	Treatment—Unfenced	0.26	0.16	1.59		
	**Forest type**—**Rainforest**	**−0.95**	**0.25**	**−3.76**		
	Forest type—Wet forest	−0.38	0.25	−1.52		
	**Treatment(Unfenced) × Time**	**−0.16**	**0.07**	**−2.12**		
	Treatment(fenced) × Time	−0.02	0.07	−0.21		
(b) Germinant count	(Intercept)	−0.87	0.36	−2.39	0.23	0.93
	**Time**	**0.10**	**0.01**	**7.00**		
	Treatment—Fenced	0.21	0.37	0.57		
	Treatment—Unfenced	0.54	0.38	1.43		
	Season—Winter	−0.33	0.19	−1.72		
	**Season**—**Spring**	**0.75**	**0.17**	**4.45**		
	**Season**—**Summer**	**−0.52**	**0.21**	**−2.53**		
	Treatment(Unfenced) × Time	−0.01	0.02	−0.54		
	**Treatment(Fenced) × Time**	**−0.09**	**0.02**	**−4.55**		
(c) Seedling count	(Intercept)	−3.13	0.57	−5.50	0.17	0.94
	**Time**	**0.15**	**0.01**	**14.06**		
	Treatment—Fenced	0.86	0.50	1.73		
	Treatment—Unfenced	0.90	0.48	1.86		
	**Season**—**Winter**	**−0.35**	**0.09**	**−3.72**		
	**Season**—**Spring**	**−0.75**	**0.10**	**−7.67**		
	**Season**—**Summer**	**−0.22**	**0.11**	**−1.98**		
	**Treatment(Unfenced) × Time**	**−0.05**	**0.01**	**−3.60**		
	Treatment(Fenced) × Time	−0.02	0.01	−1.78		

## APPENDIX D

Model outputs for the relationship between experimental treatments and vegetation structure. Models are GLMMs (assuming a binomial error distribution). In all models, “simulated” was the reference category for treatment to allow for comparison between potential engineering effects (simulated c.f. fenced) and herbivory effects (simulated c.f. unfenced). Autumn was the reference category for season. “*” denotes interactions between fixed effects. Significant effects are shown in bold

**Table:**

Response	Fixed effect	Estimate	SE	*z* value	*R* ^2^_(marginal)_	*R* ^2^_(conditional)_
(a) Vegetation Structure 0–0.5 m (touches on range pole)	(Intercept)	−2.80	0.49	−5.71	0.05	0.37
	Time	0.03	0.01	1.94		
	Treatment—Unfenced	0.21	0.56	0.38		
	Treatment—Fenced	0.48	0.55	0.88		
	Vegetation type—Rainforest	−0.13	0.48	−0.27		
	Vegetation type—Wet forest	−0.32	0.48	−0.67		
	**Treatment(Unfenced) × Time**	**−0.04**	**0.02**	**−1.97**		
	Treatment(Fenced) × Time	0.01	0.02	0.74		
(b) Vegetation Structure 0.5–1 m (touches on range pole)	(Intercept)	−3.52	0.64	−5.53	0.05	0.39
	Time	−0.01	0.02	−0.49		
	Treatment—Unfenced	0.05	0.64	0.07		
	Treatment—Fenced	1.02	0.59	1.72		
	Vegetation type—Rainforest	−0.35	0.64	−0.55		
	Vegetation type—Wet forest	−0.38	0.64	−0.59		
	Treatment(Unfenced) × Time	−0.01	0.03	−0.29		
	Treatment(Fenced) × Time	−0.01	0.03	−0.24		
(c) Vegetation Structure 1–1.5 m (touches on range pole)	(Intercept)	−4.98	0.91	−5.48	0.23	0.53
	Time	−0.13	0.07	−1.90		
	Treatment—Unfenced	1.18	0.83	1.43		
	Treatment—Fenced	0.48	0.86	0.56		
	Vegetation type—Rainforest	0.50	0.80	0.62		
	Vegetation type—Wet forest	0.15	0.82	0.19		
	Treatment(Unfenced) × Time	0.11	0.07	1.52		
	Treatment(Fenced) × Time	0.14	0.07	1.89		
(d) Vegetation Structure 1.5–2 m (touches on range pole)	(Intercept)	−3.81	0.58	−6.61	0.16	0.41
	**Time**	**−0.08**	**0.03**	**−2.63**		
	**Treatment**—**Unfenced**	**1.27**	**0.55**	**2.31**		
	Treatment—Fenced	0.75	0.56	1.34		
	**Vegetation type**—**Rainforest**	**1.17**	**0.55**	**2.14**		
	Vegetation type—Wet forest	0.55	0.55	0.99		
	Treatment(Unfenced) × Time	0.03	0.03	1.06		
	Treatment(Fenced) × Time	0.02	0.03	0.70		

## APPENDIX E

Model outputs (LMMs assuming Gaussian error distribution) for the relationship between experimental treatments and herb and ground fern cover. “Simulated” was the reference category for treatment, to allow for comparison between potential engineering effects (simulated *c.f*. fenced) and herbivory effects (simulated *c.f*. unfenced). “*” denotes interactions between fixed effects. Significant predictors are presented in bold

**Table:**

Response	Fixed effect	Estimate	SE	*t* value	*R^2^*_(marginal)_	*R^2^*_(conditional)_
(a) Herbs (% cover, Logit‐transformed)	(Intercept)	−3.54	0.44	−8.08	0.10	0.90
	**Time**	**0.03**	**0.01**	**4.13**		
	Treatment—Unfenced	0.21	0.32	0.65		
	Treatment—Fenced	−0.09	0.32	−0.26		
	Vegetation type—Rainforest	−0.93	0.56	−1.67		
	Vegetation type—Wet forest	−0.21	0.56	−0.37		
	**Treatment_(Unfenced)_ × Time**	**−0.03**	**0.01**	**−3.47**		
	Treatment_(Fenced)_ * Time	0.00	0.01	0.15		
(b) Ground ferns (% cover, Logit‐transformed)	(Intercept)	−3.32	0.46	−7.22	0.03	0.85
	Time	0.00	0.01	−0.12		
	Treatment—Unfenced	−0.28	0.35	−0.80		
	Treatment—Fenced	−0.12	0.35	−0.34		
	Vegetation type—Rainforest	0.36	0.48	0.74		
	Vegetation type—Wet forest	−0.16	0.48	−0.33		
	Treatment_(Unfenced)_ * Time	0.00	0.01	0.25		
	Treatment_(Fenced)_ * Time	0.01	0.01	1.05		
